# The significance of *Lactobacillus crispatus* and *L. vaginalis* for vaginal health and the negative effect of recent sex: a cross-sectional descriptive study across groups of African women

**DOI:** 10.1186/s12879-015-0825-z

**Published:** 2015-03-04

**Authors:** Vicky Jespers, Janneke van de Wijgert, Piet Cools, Rita Verhelst, Hans Verstraelen, Sinead Delany-Moretlwe, Mary Mwaura, Gilles F Ndayisaba, Kishor Mandaliya, Joris Menten, Liselotte Hardy, Tania Crucitti, for the Vaginal Biomarkers Study Group

**Affiliations:** Department of Public Health, Unit of Epidemiology and Control of HIV/STD, Institute of Tropical Medicine, Nationalestraat 155, B-2000 Antwerp, Belgium; Department of Clinical Infection, Microbiology and Immunology, Institute of Infection and Global Health, University of Liverpool, Liverpool, UK; Laboratory Bacteriology Research, University Gent, Ghent, Belgium; International Center for Reproductive Health (ICRH), Ghent University, Ghent, Belgium; Department of Obstetrics and Gynaecology, Faculty of Medicine and Health Sciences, Ghent University, Ghent, Belgium; Wits Reproductive Health & HIV Institute, University of Witwatersrand, Johannesburg, South Africa; ICRH Kenya, Mombasa, Kenya; Rinda Ubuzima, Kigali, Rwanda; Department of Clinical Sciences, Institute of Tropical Medicine, Antwerp, Belgium; Department of Clinical Sciences, HIV/STI Reference Laboratory, Institute of Tropical Medicine, Antwerp, Belgium

**Keywords:** Bacterial vaginosis, *Lactobacillus*, reproductive health, Sexually transmitted infections, quantitative PCR, Sub-Saharan Africa, Vaginal microbiota, Sexual health, HIV prevention

## Abstract

**Background:**

Women in sub-Saharan Africa are vulnerable to acquiring HIV infection and reproductive tract infections. Bacterial vaginosis (BV), a disruption of the vaginal microbiota, has been shown to be strongly associated with HIV infection. Risk factors related to potentially protective or harmful microbiota species are not known.

**Methods:**

We present cross-sectional quantitative polymerase chain reaction data of the *Lactobacillus* genus, five *Lactobacillus* species, and three BV-related bacteria (*Gardnerella vaginalis*, *Atopobium vaginae*, and *Prevotella bivia*) together with *Escherichia coli* and *Candida albicans* in 426 African women across different groups at risk for HIV. We selected a reference group of adult HIV-negative women at average risk for HIV acquisition and compared species variations in subgroups of adolescents, HIV-negative pregnant women, women engaging in traditional vaginal practices, sex workers and a group of HIV-positive women on combination antiretroviral therapy. We explored the associations between presence and quantity of the bacteria with BV by Nugent score, in relation to several factors of known or theoretical importance.

**Results:**

The presence of species across Kenyan, South African and Rwandan women was remarkably similar and few differences were seen between the two groups of reference women in Kenya and South Africa. The Rwandan sex workers and HIV-positive women had the highest *G. vaginalis* presence (p = 0.006). Pregnant women had a higher *Lactobacillus* genus mean log (7.01 genome equivalents (geq)/ml) compared to the reference women (6.08 geq/ml). *L. vaginalis* (43%) was second to *L. iners* (81.9%) highly present in women with a normal Nugent score. Recent sexual exposure negatively affected the presence of *L. crispatus* (<0.001), *L. vaginalis* (p = 0.001), and *Lactobacillus* genus (p < 0.001). Having more than one sexual partner in the last three months was associated with an increased prevalence of *G. vaginalis* (p = 0.044) and *L. iners* (p = 0.001).

**Conclusions:**

Although the composition of species across the studied African countries was similar, the presence of protective species i.e. *L. crispatus* and *L. vaginalis* in women with a normal Nugent score appeared lower compared to non-African studies. Furthermore, *Lactobacillus* species were negatively affected by sexual behavioural. Strategies to support protective *Lactobacillus* species are urgently needed.

**Trial registration:**

The study is registered at the Trial Registration at the National Health Research Ethics Council South Africa with the number DOH2709103223.

**Electronic supplementary material:**

The online version of this article (doi:10.1186/s12879-015-0825-z) contains supplementary material, which is available to authorized users.

## Background

Bacterial vaginosis (BV) has been consistently associated with an increased risk of HIV infection and other sexually transmitted infections (STI) [1] as well as adverse clinical outcomes such as pelvic inflammatory disease [2], miscarriage [3], septic postpartum and neonatal infections [4]. BV can increase the risk of other STI which are in turn associated with HIV acquisition, including human papilloma virus and herpes simplex virus type 2 (HSV-2) infection [5-7]. While BV is a common condition worldwide, the highest prevalence is seen in sub-Saharan countries where HIV prevalence is highest [8]. BV is best described as a disruption of the vaginal microbiota or as a vaginal bacterial dysbiosis. It is characterised by a reduction or a replacement of the protective *Lactobacillus* species by an overgrowth of other anaerobic bacteria [9,10].

Advances in molecular technologies, such as polymerase chain reaction (PCR) based techniques, have provided us with new insights and a more detailed characterisation of the vaginal microbiota; however, the aetiology of BV remains poorly understood. Data on the composition of the vaginal microbiota of African populations from regions with generalised HIV epidemics is still very limited. In order to design efficient biomedical interventions we need a better knowledge of the variations of the vaginal microbiota in healthy women at average risk of HIV infection and in women at high risk of HIV infection [11]. Further research is needed to address how the composition of the vaginal microbiota determines optimal vaginal and reproductive health. This knowledge may advance the development of novel interventions to prevent new HIV infections and address biological vulnerability in young adolescent women.

Our study examined individual vaginal bacterial species in women in sub-Saharan Africa and investigated correlations with vaginal health and associated factors. This paper will report cross-sectional quantitative PCR data of the *Lactobacillus* genus and the five most prevalent vaginal *Lactobacillus* species [12,13] as well as three BV-related bacteria (*Gardnerella vaginalis*, *Atopobium vaginae*, and *Prevotella bivia),* and *Escherichia coli* and *Candida albicans* in groups of women in sub-Saharan Africa. To address the research gap described above we studied the presence and concentrations of the species in women at different risk for HIV infection. We quantified the species in a reference group of adult HIV-negative women at average risk for HIV acquisition and compared species variations in subgroups of HIV-negative pregnant women, adolescents, women engaging in intravaginal practices, sex workers and a group of HIV-positive women on combination antiretroviral therapy. We explored the associations between presence and concentrations of the bacteria with country, group, and in relation to several factors of known or theoretical importance e.g. recent sexual exposure including a seminal plasma biomarker [14], current contraceptive use, and reproductive tract infections (RTI).

## Methods

### Study design

A total of 430 women were enrolled at three study sites in Mombasa, Kenya (KE), Kigali, Rwanda (RW) and Johannesburg, South Africa (SA) in 2010–2011 [15]. Participants were enrolled in the cohort in one of six pre-defined study groups: a reference group of 219 non-pregnant HIV-negative women (KE 110; SA 109), and pregnant women (KE 30; SA 30), adolescents (KE 30; SA 30), women practicing intravaginal practices (SA 30), self-declared sex workers (RW 30) or HIV-positive women (RW 30).

### Population

The reference group consisted of women (18–35 years) who were at average risk for HIV acquisition, did not engage in traditional intravaginal practices and were not pregnant. The pregnant women were less than 14 weeks gestation. The non-pregnant adolescents were 16 or 17 years old. The non-pregnant women engaging in traditional intravaginal practices inserted substances (cloth/lemon juice/detergents) other than water and/or fingers to clean, dry or tighten the vagina on a regular basis. The remaining HIV-negative women consisted of non-pregnant self-declared sex workers. The HIV-positive women were on antiretroviral treatment for at least 6 months, were currently asymptomatic and had a CD4 count of more than 350 cells/μl. Recruitment was as follows: healthy women for inclusion in the reference groups were recruited primarily through women’s groups and subsequent snowballing in Mombasa County and through primary health care clinics in inner city Johannesburg. Women practicing intra-vaginal practices were identified as a sub-set of this group. Pregnant women were recruited at antenatal clinics in Mombasa County and inner city Johannesburg. Adolescents were recruited from youth groups in Mombasa County and from youth-friendly clinics in inner city Johannesburg. In Kigali, sex workers were recruited from the sex worker community and from a previous prospective HIV-incidence cohort at RU [16,17], using community mobilizers; and HIV-positive women were recruited from the Muhima hospital public HIV treatment clinic.

### Clinic visit and laboratory procedures

The screening and eligibility assessment included testing for HIV infection, RTI (*Chlamydia trachomatis* (CT), *Neisseria gonorrhoea* (NG), *Trichomonas vaginalis* (TV), HSV-2 and syphilis, urinary tract infection and cervical dysplasia by Pap smear. Women were enrolled on day 9 (+/− 2 days) of the menstrual cycle with a maximum of two months after the screening visit. All visits were conducted by a qualified person and done in the language chosen by the participant. An interview was conducted on sexual behaviour and vaginal practices. Next, a physical and vaginal speculum examination was carried out by a clinician, including colposcopy. Vaginal swabs for quantitative polymerase chain reaction (qPCR) analysis were taken at the enrolment visit before any other samples to avoid contamination. More details on visits and procedures have been described previously [15]. Vaginal swabs (Copan Diagnostics, Inc., Murrieta, USA) were shipped in batches using a temperature-monitored dry shipper to the central laboratory at the Institute of Tropical Medicine (ITM) in Antwerp, Belgium. Vaginal Gram-stained smears were scored at the ITM using the Nugent method in which a Nugent score of 7–10 is classified as positive for BV, 4–6 is classified as intermediate and 0–3 indicates a normal vaginal microbiota [18]. For the quantification of vaginal species, two vaginal swabs per woman were taken and stored at – 80°C until DNA extraction. The swabs were thawed for 30 minutes at room temperature and diluted phosphate buffered saline (PBS) (1,200 μl; 1 part PBS and 9 parts saline, pH 7.4) was added to each swab and vortexed for 15 seconds. One ml of each suspension was pooled to a final volume of 2 ml. An aliquot of 250 μl was used for DNA extraction on the Abbott *m*24*sp* automated extraction platform (Abbott, Maidenhead, United Kingdom) according to the manufacturer’s instructions. 200 μl of eluted DNA was stored at −80°C for the qPCR assays (Additional file 1). The eluted swab suspension was tested for the presence of prostate-specific antigen (PSA) using the Seratec PSA semiquant assay (Seratec Diagnostica, Göttingen, Germany). A volume of 150 μl of the eluted swab suspension was centrifuged for 10 min at 13,000 × g. After centrifugation, 120 μl of supernatant was used for testing according to the manufacturer’s instruction. The vaginal pH was measured using pH 3.6-6.1 paper strips pressed against the vaginal wall during the pelvic examination (Macherey-Nagel pH Fix 3.6-6.1, Düren, Germany). The RTI diagnostic tests used in this study have been described before [15].

### Quantitative PCR of selected organisms

We designed or selected primers targeting the following genus and species which have previously been shown to be important members of the vaginal microbiota [19-22]: *Lactobacillus* genus, *L. crispatus, L. iners, L. jensenii*, *L. gasseri, L. vaginalis*, *Gardnerella vaginalis*, *Atopobium vaginae*, *Prevotella bivia*, *E. coli* [23], and *C. albicans* [10]. qPCR was performed at the ITM and at the University of Ghent, Ghent, Belgium, as follows: at the ITM, for *Lactobacillus* genus, *L. crispatus, L. iners, L. jensenii*, *L. gasseri,* and *L. vaginalis*, the 25 μl PCR mixture contained 12.5 μl Rotor-Gene SYBR Green RT-PCR Master mix (Rotor-Gene SYBR Green PCR Kit, Qiagen, Venlo, The Netherlands), 5 μl DNA extract, 0.5-1.0 μM of their respective primers (Integrated DNA Technologies, Leuven, Belgium), and RNase-Free Water provided with the Rotor-Gene SYBR Green PCR kit [13,21,22,24,25]. The amplification reactions were performed with the Rotor Gene Q MDx 5 plex (Qiagen, Venlo, The Netherlands). The qPCR reactions, at the University of Ghent, for *A. vaginae, G. vaginalis, P. bivia* and *E. coli* were performed in a reaction volume of 10 μl, containing 5 μl of LightCycler 480R SYBR Green I Master (Roche Applied Science, Basel, Switzerland), 0.2-1.25 μM of their respective primers (Eurogentec, Liege, Belgium) and 2 μl of DNA extract [19,22,26,27]. Amplification was carried out on the LightCycler480® and the LightCyclerR 480 Software Version 1.5 (Roche, Basel, Switzerland). Standard curves were constructed for each of the organisms with 6 standards by a tenfold dilution of the DNA stock in HPLC grade water. DNA of the lactobacilli was extracted from cultures of *L. crispatus* LMG 9479^T^, *L. gasseri* LMG 9203^T^, *L. iners* LMG 18914^T^, *L. jensenii* LMG 6414^T^ and *L. vaginalis* LMG 12891^T^ grown at 35°C ±2°C on Columbia agar base (BBL, Becton Dickinson, Erembodegem, Belgium) and 5% horse blood under anaerobic conditions (Anaerocult A, Merck, VWR International, Leuven). The DNA was extracted from cultures of *A. vaginae* CCUG 38953^T^, *G. vaginalis* ATCC14018^T^*, E. coli* ACM1803^T^ grown on TSA and 5% sheep blood (Becton Dickinson, Erembodegem, Belgium) and *P. bivia* ATCC29303^T^ grown on Columbia agar (Becton Dickinson, Erembodegem, Belgium) at 37°C ±2°C under anaerobic conditions (BugBox, LedTechno, Heusden-Zolder, Belgium). After extraction, the DNA concentrations were determined using NanoDrop (Thermo Fisher scientific, Erembodegem, Belgium). The genomic concentrations were calculated using the described genomic sizes of the type strains. Both the standard curves and samples were run in duplicate. The number of bacteria was expressed as genome equivalents per ml (geq/ml).

### Data analysis

Data analysis was performed using SAS 9.4 and R 3.0.1. Each participant contributed a single data point for each analysis. Data from the enrolment visit was used except for RTI diagnoses and information about sex partners, which was collected at screening. The study population characteristics and reproductive health and vaginal microbiota data (including vaginal pH, Nugent scores, and qPCR bacterial presence and concentrations in log geq/ml) are described as medians with ranges for continuous variables and concentrations and percentages for categorical variables. qPCR data was expressed categorically as presence/absence, or alternatively presence was divided into three separate categories: Not quantifiable (1600 to 16.000 geq/ml), <10^6^ geq/ml, ≥10^6^ geq/ml. For quantifiable levels, mean and SD were calculated. A positive PSA result included both strong and weak reactions. We explored bivariate associations with chi-squared tests of the presence/absence of each bacterial species (*Lactobacillus* genus excluded) with the following variables: country, group, age, parity, lifetime number of sexual partners, number of sexual partners in the last three months, reported recent vaginal sex, seminal factor PSA presence, colposcopic findings e.g. petechiae, erythema, presence of ectopy, HSV-2 serology, RTI (excluding HIV, HSV-2, Candida), products used to wash/cleanse/dry/tighten, intravaginal cleansing during bathing, contraception, recent antibiotic use (excluding cotrimoxazole prophylaxis for the prevention of HIV-associated opportunistic infections), BV, and vaginal pH. For *Lactobacillus* genus we performed a simple linear regression analysis including the variables defined above. In the multivariate logistic regression, we report adjusted odds ratio (AOR) and 95% confidence intervals (CI); all variables meeting a p-value of ≤0.05 in the bivariate analysis were included, removing variables with a p-value of <0.05 in a stepwise manner. For *Lactobacillus* genus we performed a multiple linear regression analysis, reported as adjusted difference in means, including the variables meeting a p-value of ≤0.05 from the simple regression analysis. We also constructed a multivariate logistic regression model that included selected species and clinically relevant variables to improve the interpretation of the data. The species and variables included in this model were: *L. crispatus*, *L. iners*, *G. vaginalis*, *A. vaginae, Lactobacillus* genus, parity, PSA, number of sexual partners within the last three months, reported intravaginal cleansing during bathing, recent antibiotic use, and contraception use. We included intravaginal cleansing during bathing as this was a highly prevalent behaviour in our study population. We further included contraceptive use in the model because the controversial discussion of the effect of progesterone depot on the acquisition of HIV. This analysis was performed on the reference group and on all women.

### Ethics statement

Written information and consent forms in the local language were provided. After the interview, the participants and, in case they were of minor age/not emancipated (age below 18 in South Africa and Kenya and below 21 in Rwanda), the parents or guardians were asked to confirm their willingness to participate in the study by signing or marking the consent form. The protocol was approved by the Kenyatta National Hospital Ethical Review Committee, Kenya; the Human Research Ethics Committee, University of the Witwatersrand, SA; the Rwanda National Ethics Committee, Rwanda; the Institutional Review Boards of the Institute of Tropical Medicine in Antwerp, of Ghent University, and of the University Teaching Hospital in Antwerp, Belgium. In addition the study was approved by the National Council on Science and Technology in Kenya; the SA Department of Health; and the National AIDS Control Commission in Rwanda.

## Results

qPCR data were not available for four women leaving 426 women for the analysis. Nugent score data were available for 387 of the 426 women due to unreadable Gram stain smears.

The mean age of the reference group was 25 years (Additional file 2). Women in the other study groups had a similar mean age (24–26 years), except for the adolescents who were 16–17 years old, and the HIV-positive women who had a mean age of 31 years. Contraceptive use in the reference group was 80.4%; the most commonly used methods were condoms (24%) and progestin-only injections (36%). Among adolescents, overall contraceptive use was low (65%); the most common method was condoms (50%). Seventy-five per cent of women in the reference group had one or more children. Parity was highest in the vaginal practices group (84%), the sex workers (97%) and HIV-positive women (90%). Intravaginal cleansing during bathing was frequently in all groups (including the reference group), with exception of pregnant women who reported less intravaginal cleansing (28%). While the majority of women stated having one sex partner in the past three months (reference group 90%), with exception of sex workers who reported many more partners. Systemic antibiotics were used by 62 women (14%) within 14 days prior to the enrolment visit. The last day of antibiotic use was on average 7 days (median 7 days) prior to the enrolment visit.

### Prevalence of BV, pH and PSA

Overall, 57.5% of women presented with a normal Nugent score, 7.1% with an intermediate score and 35.4% with a BV score. The BV prevalence was 33% in the reference group, 30% for pregnant women, 30% in adolescents, 37% in vaginal practice users, 48% for women living with HIV, and 68% for sex workers. The overall mean vaginal pH was 4.7 (SD = 0.7) with the majority (68%) of women having a pH between four and five (Additional file 3). Ten per cent of women had a pH below four. PSA was present for 39% of the reference group, 38% in the adolescents, 44% among women living with HIV, 45% in the vaginal practices users, 56% in pregnant women, and 57% in sex workers. Further results by group are presented in Table 1.

**Table 1:** **Presence and concentrations of vaginal microbiota species**

	**Reference group**		**Pregnant women**		**Adolescents**		**Intravaginal practices**	**Sex workers**	**HIV-positive**
	**Kenya N = 109**	**South Africa N = 108**	**Kenya N = 30**	**South Africa N = 30**	**Kenya N = 29**	**South Africa N = 30**	**South Africa N = 30**	**Rwanda N = 30**	**Rwanda N = 30**
***Presence of species by qPCR***	N (%)	N (%)	N (%)	N (%)	N (%)	N (%)	N (%)	N (%)	N (%)
*Lactobacillus* genus	101(93)	98(91)	30(100)	30(100)	27(93)	24(80)	28(93)	28(93)	24(80)
*Lactobacillus crispatus*	29(27)	26(24)	6(20)	7(23)	11(38)	6(20)	5(17)	5(17)	5(17)
*Lactobacillus iners*	75(69)	82(76)	24(80)	23(77)	23(79)	20(67)	26(87)	18(60)	19(63)
*Lactobacillus jensenii*	19(17)	25(23)	6(20)	10(23)	7(24)	2(7)	6(20)	1(3)	3(10)
*Lactobacillus gasseri*	7(6)	8(7)	3(10)	3(10)	4(14)	2(7)	2(7)	4(13)	3(10)
*Lactobacillus vaginalis*	33(30)	28(26)	9(30)	7(23)	14(48)	7(23)	5(17)	8(27)	9(30)
*Gardnerella vaginalis*	59(54)	49(45)	14(47)	15(50)	17(59)	19(63)	16(53)	23(73)	21(70)
*Atopobium vaginae*	46(42)	37(34)	9(30)	11(37)	10(34)	19(43)	11(37)	17(57)	14(47)
*Prevotella bivia*	97(89)	83(77)	29(97)	21(70)	24(83)	12(40)	22(63)	30(100)	27(70)
*Escherichia coli*	28(26)	29(27)	6(20)	10(23)	8(28)	4(13)	9(30)	21(70)	6(20)
*Candida albicans*	15(14)	12(11)	4(13)	1(3)	3(10)	0	5(17)	4(13)	3(10)
***Vaginal species concentrations by qPCR*** ^***1***^	Mean (SD)	Mean (SD)	Mean (SD)	Mean (SD)	Mean (SD)	Mean (SD)	Mean (SD)	Mean (SD)	Mean (SD)
*Lactobacillus* genus	6.4(2.1)	6.9(1.7)	7.5(1)	6.5(2.3)	7.5(1.1)	5.3(2.5)	6.4(2.1)	7.1(1.7)	6.5(2.4)
*Lactobacillus crispatus*	6.8(2.2)	7.2(1.6)	8(0.4)	7.6(1.5)	7.3(2.2)	7.1(1.3)	7.5(1.3)	7.8(1.1)	4.7(3.4)
*Lactobacillus iners*	6.6(2.2)	7(2)	7.6(1.1)	6.9(1.6)	7.4(1.7)	4.9(3.1)	6.7(2.3)	8.1(1.1)	7.3(2.4)
*Lactobacillus jensenii*	5.6(2.5)	6.3(1.9)	6.6(1)	7.7(0.9)	5.5(3.1)	3.5(3.6)	5(3.2)	8.3(NA)	6.3(1)
*Lactobacillus gasseri*	3.3(2.9)	5.3(1.9)	6.3(1.3)	5.1(3.8)	4.7(2.5)	6.4(0.4)	3.8(4)	4.1(3.5)	1(NA)
*Lactobacillus vaginalis*	2.6(3)	3.8(3.2)	3.9(3.4)	4.4(3.9)	5.2(2.8)	6.2(0.7)	3.1(4.4)	4.8(3.2)	4.1(2.7)
*Gardnerella vaginalis*	5.4(1.1)	5.3(0.9)	5.5(1.2)	5.2(0.9)	5.5(0.8)	4.6(1.2)	5.2(1.4)	6.2(1)	5.6(1)
*Atopobium vaginae*	5.5(1.8)	5.1(1.5)	7(1)	5.2(1.7)	5.4(2.4)	5(1.7)	5.7(1.3)	6.6(1.8)	5.5(1.5)
*Prevotella bivia*	3.1(1.2)	3.2(1.1)	3.1(1.3)	3.3(0.9)	3.3(1.3)	3.2(1.1)	3.5(1.1)	3.6(1.1)	3.3(1)
*Escherichia coli*	5.2(0.4)	5.1(0.6)	5(0.5)	5.2(0.5)	5.1(0.4)	4.8(0.3)	4.9(0.3)	5.3(0.6)	5.9(1.1)
*Candida albicans*	4.9(1.3)	5.1(0.7)	5.7(1)	4.6(NA)	6(0.7)	0(NA)	5.3(0.8)	5.4(1.2)	4.9(0.2)
***Nugent score*** ^***2***^ ***and vaginal pH***									
Vaginal pH	5.1(0.9)	4.5(0.6)	4.8(0.8)	4.3(0.5)	4.7(0.7)	4.4(0.6)	4.6(0.6)	5.1(0.6)	4.7(0.6)
Nugent 0–3: normal	55(56)	63(66)	19(68)	18(62)	18(67)	13(45)	16(59)	6(24)	13(48)
Nugent 4–6: intermediate	8(8)	6(6)	1(4)	2(7)	4(15)	4(14)	1(4)	2(8)	1(4)
Nugent 7–10: bacterial vaginosis	36(36)	27(28)	8(29)	9(31)	5(19)	12(41)	10(37)	17(68)	13(48)

Quantitative PCR data was available for 426 of the 430 women. ^1^mean (SD) quantity log concentrations of the microbiota for women who had that particular species detected and quantified as log genome equivalents/ml. ^2^Nugent score data were available for 387 of the 426 women due to unreadable Gram stained slides. qPCR: quantitative polymerase chain reaction.

### A comparison of the presence and concentrations of species and *Lactobacillus* genus across countries and groups

**Figure 1:**
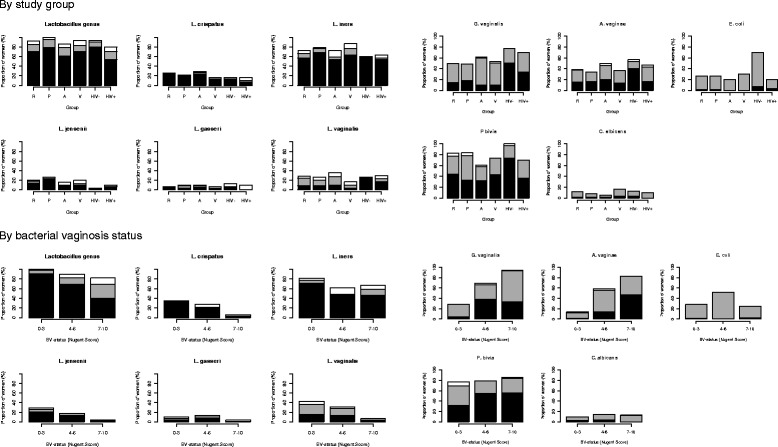
**Presence of microbiota species by study group and bacterial vaginosis status.** R: reference group; P: pregnant women; A: adolescent women; V: women using vaginal practices other than water and soap; HIV-: self-declaring sex workers; HIV+: HIV-positive women. Black bar: ≥10^6^ genome equivalents (geq)/ml; Grey bar: <10^6^ geq/ml (10^3^ geq/ml for *P. bivia*); White bar: present but not quantifiable (1600 to 16.000 geq/ml).

The presence of species and *Lactobacillus* genus across Kenyan, South African and Rwandan women was remarkably similar and there was no evidence of differences between the two large groups of reference women in Kenya and South Africa (Figure 1, Tables 1 and 2, Additional file 3). There was strong evidence for differences among the Rwandan sex worker group. The Rwandan sex workers had the highest *G. vaginalis* presence (p = 0.006) and the lowest *L. jensenii* presence (6.7% RW, 19% KE, 21.7% SA; p = 0.031). Also, *E. coli* was present in 70% of Rwandan sex workers compared to 20% in Rwandan HIV-positive women, 25% in women in Kenya and 26% in SA (p = 0.009). High concentrations of *Lactobacillus* genus (>10^6^ geq/ml) were present in 69% of all women and low concentrations (10^3^ to 10^6^ geq/ml) in 15% (a distribution of the species log concentrations is shown in Additional file 4). *L. iners, L. crispatus, L. jensenii, L. vaginalis,* and *L. gasseri* were detected in high concentrations (>10^6^ geq/ml) in 58%, 20%, 13%, 10% and 3% of women, respectively. In the reference group 91.7% of women had lactobacilli detected as measured by *Lactobacillus* genus-level PCR. This proportion was lowest for HIV-positive women (80%) and for adolescents (86.4%). The *Lactobacillus* genus mean log was 6.39 geq/ml for Kenyan women which was higher than the mean log for Rwandan (5.93 geq/ml) and South African (5.95 geq/ml) women (p = 0.235). Pregnant women, in particular in Kenya, had a higher mean log of *Lactobacillus* genus (7.01 geq/ml) compared to the reference women (6.08 geq/ml; p = 0.013). Interestingly, South African adolescents had the lowest mean log *Lactobacillu*s genus (4.26 geq/ml, p < 0.001) whereas Kenyan adolescent had a high mean log of 6.97 geq/ml when comparing groups and country. Further, adolescents had low *P. bivia* presence compared to the reference group (p < 0.001). *C. albicans* was detected in 12% of reference women and similar proportions were found in the other groups (p = 0.524).

**Table 2:** **Univariate and multivariate analysis of factors associated with vaginal** ***Lactobacillus*** **, and BV-related species presence** ^**1**^

**N = 426**		***L. crispatus***		***L. iners***		***L. vaginalis***		***G. vaginalis***		***A. vaginae***		***P. bivia***
	%	OR (CI)	%	OR (CI)	%	OR (CI)	%	OR (CI)	%	OR (CI)	%	OR (CI)
**Country**		p = 0.207		p = 0.084		p = 0.126		p = 0.006		p = 0.182		**p = <0.001**
*Kenya*	27.4		72.6		33.3		53.6	Ref	38.7		89.3	**Ref**
*Rwanda*	16.7		61.7		28.3		73.3	2.38(1.25,4.55)	51.7		85.0	**0.68(0.29,1.61)**
*South Africa*	22.2		76.3		23.7		50.0	0.87(0.57,1.31)	39.4		69.7	**0.28(0.16,0.49)**
**Group**		p = 0.580		p = 0.174		p = 0.591		p = 0.026		p = 0.190		p = <0.001
*Reference group*	25.3		72.4		28.1		49.8	Ref	38.2		82.9	Ref
*Pregnant women*	21.7		78.3		26.7		48.3	0.94(0.53,1.67)	33.3		83.3	1.03(0.48,2.21)
*Adolescents*	28.8		72.9		35.6		61.0	1.58(0.88,2.84)	49.2		61.0	0.32(0.17,0.61)
*Intravaginal practices*	16.7		86.7		16.7		53.3	1.15(0.54,2.48)	36.7		73.3	0.57(0.23,1.37)
*Sex workers*	16.7		60.0		26.7		76.7	3.32(1.37,8.05)	56.7		100	No estimate
*HIV-positive women*	16.7		63.3		30.0		70.0	2.35(1.03,5.37)	46.7		70.0	0.48(0.2,1.37)
**Age**		p = 0.376		p = 0.046		p = 0.554		p = 0.640		p = 0.556		p = <0.001
*<18 years*	28.8		72.9	Ref	35.6		61.0		49.2		61.0	Ref
*18 – 24 years*	20.3		77.0	1.25(0.63,2.49)	25.7		56.1		40.5		84.5	3.98(1.93,8.24)
*25 – 29 years*	21.7		75.4	1.14(0.57,2.27)	27.5		52.2		39.1		83.3	3.37(1.62,7.03)
*30 years or more*	28.4		60.5	0.57(0.28,1.18)	28.4		51.9		38.3		77.8	2.46(1.08,5.58)
**Parity**		p = 0.106		p = **0.001**		p = **0.03**		p = 0.418		p = 0.840		p = 0.164
*0*	27.5		76.5	**Ref**	34.2	**Ref**	54.4		40.3		75.2	
*1 – 2*	19.2		75.7	**0.96(0.59,1.56)**	22.4	**0.56(0.35,0.89)**	57.0		42.1		83.2	
*>2*	28.6		54.0	**0.36(0.19,0.67)**	33.3	**0.96(0.51,1.79)**	47.6		38.1		77.8	
**RTI** ^**2**^		p = **0.014**		p = 0.406		p = 0.031		p = 0.019		p = **0.003**		p = 0.977
*No RTI*	25.8	**Ref**	72.0		30.3	Ref	52.1	Ref	37.7	**Ref**	79.6	
*1 or more RTI*	12.3	**0.40(0.19,0.85)**	76.7		17.8	0.50(0.26,0.95)	67.1	1.88(1.10,3.19	56.2	**2.12(1.27,3.53)**	79.5	
**Nr of sexual partners last 3 months** ^**3**^		p = 0.060		p = **<0.001**		p = 0.669		p = **0.033**		p = 0.129		p = 0.053
*0*	42.3		42.3	**Ref**	30.8		34.6	**Ref**	30.8		76.9	Ref
*1*	22.5		75.8	**4.27(1.89,9.64)**	27.3		54.6	**2.28(0.99,5.24)**	40		78.0	1.07(0.41,2.74)
*>1*	20.0		66.7	**2.73(1.01,7.37)**	33.3		66.7	**3.78(1.36,10.46)**	53.3		93.3	4.2(0.95,18.54)
**Seminal factor PSA present** ^**4**^		p = **<0.001**		p = 0.464		p = **<0.001**		p = 0.259		p = 0.308		p = 0.128
*No*	32.1	**Ref**	70.9		34.6	**Ref**	52.1		38.5		82.9	
*Yes*	13.2	**0.32(0.19,0.54)**	74.2		19.8	**0.47(0.3,0.73)**	57.7		43.4		76.9	

Results that remained significant (using a p = 0.05 cut-off) in the multivariate logistic regression are depicted in bold. ^1^: Univariate analysis with p-values from chi-squared tests; ^2^RTI: excluding HIV, HSV-2, Candida**.** ^3^Data collected at the screening visit up to two months before the enrolment visit. ^4^Prostate specific antigen present in vaginal fluid, including weak reaction. RTI: reproductive tract infections.

### Reproductive health, sexual behaviour and the presence and concentrations of qPCR microbiota

Parity was negatively associated with *L. vaginalis* and *L. iners* in a bimodal way. Having one or two children was associated with a lower prevalence of *L. vaginalis* (22.4%) compared to women with no children (34.2%) and women with more than two children (33.3%) (AOR 0.56; 95% CI 0.35,0.91; p = 0.05). This association was similar for the reference group (20% vs 37.9% vs 38.5%; AOR 0.41; 95% CI 0.20, 0.82). Further, having more than two children was associated with lower detection of *L. iners* (AOR 0.35; 95% CI 0.18,0.66; p = 0.002). Finally, having any number of children was strongly associated with the presence of *C. Albicans* (AOR 3.7; 95% CI 1.59, 8.61 for 1–2 children and AOR 2.54; 95% CI 0.85,7.56 for more than 2 children; p = 0.006). We found several sexual behavioural factors associated with presence and concentrations of species. *L. crispatus* (AOR 0.33; 95% CI 0.20,0.56; p < 0.001) and *L. vaginalis* presence (AOR 0.47; 95% CI 0.3,0.75;p = 0.001) was negatively associated with recent sexual exposure as measured by PSA detection, as were the *Lactobacillus* genus concentrations (adjusted difference in means −1.01; 95% CI:-1.51,-0.51; p < 0.001). This strongly negative association of *L. crispatus* and PSA remained in the sub-analysis of the reference group (AOR 0.35; 95% CI 0.17,0.72; p = 0.002)*.* Additionally, having more than one sexual partner in the last three months was positively associated with the presence of *E. coli* (51.1% vs 23.1%; OR 3.48, 95% CI 1.18,10.30; p = 0.003), *L. iners* (66.7% vs 42.3%; AOR 3.1, 95% CI 1.12, 8.57; p = 0.001) and *G. vaginalis* (66.7% vs 34.6%; AOR 3.57, 95% CI 1.28,9.97; p = 0.045). There was no evidence of an association between the number of lifetime partners and the presence of species except for a modest positive association with the presence of *P. bivia* and *E. coli*. The reporting of recent vaginal sex, similarly, was only associated with *P. bivia*.

The detection of HSV-2 antibodies was negatively associated with *L. crispatus* in all women; additionally HSV-2 was negatively correlated with *Lactobacillus* genus (mean log −0.74; 95% CI: −1.46,-0.02; p = 0.045) in the reference group. The detection of an RTI at screening (CT, NG, TV, or syphilis), was negatively associated with *L. crispatus*, *L. jensenii* (9.6% vs 20.4%) and *L. vaginalis,* and positively associated with *G. vaginalis* and *A. vaginae* presence. None of the variables: abnormal colposcopic findings, products used to wash/cleanse/dry/tighten, or cleansing during bathing, showed an association with the species. The presence of ectopy was associated with increased *E. coli* presence (35.9% vs 22%; AOR 1.78, 95% CI 1.12,2.83; p = 0.002). Additionally, reported antibiotics use in the past 14 days was associated with a higher presence of *G. vaginalis* (AOR 2.23; 95% CI 1.33,3.74; p = 0.035) and *A. vaginae* (AOR 1.79; 95% CI 1.11,2.90; p = 0.017). And finally, *C. albicans* was more often present in women using progesterone-only (OR 1.96; 95% CI 1.0,3.86) or combined hormones (OR 2.47; 95% CI 1.06,5.81; p = 0.050).

### Association with Nugent score and pH

As expected, there was a strong positive association between *L. crispatus* (34.8% vs 5.8%), *L. jensenii* (28.5% vs 4.4%), *L. vaginalis* (43% vs 6.6%), *L. iners* (81.9% vs 67.2%) and *L. gasseri* (10.4% vs 3.6%) and a Nugent score 0–3 compared to a Nugent score 7–10 in both the reference group (p < 0.001 to p = 0.023) and in all women (p < 0.001 to p = 0.042) (Figure 1). Similarly, there was a strong positive association between *A. vaginae* (94.2% vs 28.1%) and *G. vaginalis* (82.5% vs 13.6%) and a Nugent score of 7–10 compared to Nugent score 0–3 in both all women and in the reference group (p < 0.001). *E. coli* presence was higher (51.7%) in women with an intermediate BV score (p = 0.011) compared to women with no BV (28.1%) and women with BV (24.1%). There was no evidence of an association between *P. bivia* and *C. albicans* and Nugent score. Vaginal pH was negatively correlated with *Lactobacillus* species, with the exception of *L. iners*. For example, *L. crispatus* was present in 67.4% of women with a pH below 4 and in 9.8% of women with a pH above 5.5 and for *L. vaginalis* proportions were 58.1% vs 11.8%. In contrast, the proportions for *L. iners* were 74.4% vs 70.6%.

### A model summarising the associations between species and relevant clinical variables

There was a negative association between *L. iners* and increased parity in both the reference women and all women (Table 3 presents OR and 95% CIs). For both the reference group and all women, the negative association between the seminal factor PSA and *L. crispatus* was confirmed, as well as a reduced *Lactobacillus* genus concentration by about one log. Similarly, for the reference group and all women, having more than one sexual partner in the last three months was positively associated with *L. iners* and *G. vaginalis* presence*.* Recent antibiotic use was associated with a higher prevalence of *G. vaginalis* and *A. vaginae* in all women, but this association was not seen in the analysis of the reference women. There was no evidence of an association with intravaginal cleansing during bathing in the analysis for all women, but there was a strong association with the microbiota in the reference women: a negative association with *A. vaginae* and positive association with *Lactobacillus* genus. In the reference group, women using progesterone-only and combined hormones had a lower presence of A*. vaginae* and *G. vaginalis* as compared to women not using contraceptives or using none hormonal contraceptive methods.

**Table 3:** **Factors associated with** ***L. crispatus, L. iners, G. vaginalis, A. vaginae*** **and** ***Lactobacillus*** **genus: results from a multiple logistic regression model for the reference women and for all women**

**Reference women N = 217**	***L. crispatus***	***L. iners***	***G. vaginalis***	***A. vaginae***	***Lactobacillus*** genus*
	OR (CI)	OR (CI)	OR (CI)	OR (CI)	Log concentrations (CI)
**Parity**	p = 0.831	p = **0.003**	p = 0.587	p = 0.265	p = 0.179
***0***	Ref	Ref	Ref	Ref	Ref
***1 – 2***	0.82(0.36,1.88)	0.43(0.18,1.05)	1.40(0.67,2.93)	1.81(0.85,3.86)	−0.79(−1.67,0.10)
***>2***	1.01(0.36,2.86)	0.16(0.05,0.47)	1.07(0.41,2.75)	1.93(0.72,5.16)	−0.91(−2.06,0.24)
**Seminal factor present**	p = **0.003**	p = 0.715	p = 0.773	p = 0.435	p = **0.016**
***No***	Ref	Ref	Ref	Ref	Ref
***Yes***	0.34(0.16,0.72)	1.13(0.58,2.23)	1.09(0.60,1.97)	1.27(0.69,2.34)	−0.89(−1.61,-0.17)
**Nr of sexual partners last 3 months** ^**1**^	p = 0.901	p = **0.049**	p = **0.049**	p = 0.204	p = 0.667
***0***	Ref	Ref	Ref	Ref	Ref
***1***	1.16(0.36,3.72)	3.90(1.27,11.94)	4.17(1.22,14.23)	2.14(0.63,7.32)	0.49(−0.84,1.83)
***>1***	0.74(0.06,9.56)	1.97(0.22,17.63)	3.12(0.34,28.91)	6.83(0.74,63.15)	−0.15(−2.74,2.44)
**Intravaginal cleansing during bathing**	p = 0.420	p = 0.208	p = 0.281	p = **0.023**	p = **0.007**
***No***	Ref	Ref	Ref	Ref	Ref
***Yes***	1.33(0.66,2.65)	1.55(0.78,3.09)	0.72(0.39,1.31)	0.49(0.26,0.91)	1.01(0.28,1.75)
**Contraception use**	p = 0.259	p = 0.368	p = **0.008**	p = **0.015**	p = 0.606
***None/Non-Hormonal***	Ref	Ref	Ref	Ref	Ref
***Hormonal***	0.66(0.32,1.36)	1.39(0.68,2.82)	0.42(0.22,0.88)	0.44(0.22,0.86)	0.21(−0.58,0.99)
**Recent antibiotic use**	p = 0.360	p = 0.704	p = 0.697	p = 0.383	p = 0.980
**No**	Ref	Ref	Ref	Ref	Ref
**Yes**	0.63(0.23,1.73)	0.83(0.33,2.13)	1.18(0.51,2.72)	1.45(0.63,3.35)	−0.01(−1.03,1.00)
**All women N = 426**	***L. crispatus***	***L. iners***	***G. vaginalis***	***A. vaginae***	***Lactobacillus*** genus*
**Parity**	p = 0.324	p = **0.002**	p = 0.477	p = 0.793	p = 0.763
***0***	Ref	Ref	Ref	Ref	Ref
***1 – 2***	0.76(0.43,1.36)	0.94 (0.54,1.66)	1.13(0.69,1.84)	1.15(0.70,1.87)	0.03(−0.58,0.64)
***>2***	1.24(0.59,2.62)	0.33 (0.16,0.67)	0.75(0.39,1.46)	0.99(0.51,1.94)	−0.19(−1.01,0.64)
**Seminal factor present**	p = **0.001**	p = 0.933	p = 0.744	p = 0.503	p = **0.001**
***No***	Ref	Ref	Ref	Ref	Ref
***Yes***	0.36(0.21,0.61)	1.01(0.63,1.61)	1.07(0.71,1.61)	1.13(0.75,1.70)	−1.00 (−1.51,-0.49)
**Nr of sexual partners last 3 months** ^**1**^	p = 0.319	p = **0.003**	p = **0.022**	p = 0.095	p = 0.310
***0***	Ref	Ref	Ref	Ref	Ref
***1***	0.50(0.21,1.17)	4.40 (1.86,10.41)	2.47 (1.02,5.98)	1.61 (0.64,4.02)	0.20(−0.85,1.25)
***>1***	0.51(0.17,1.58)	3.05 (1.06,8.79)	4.35 (1.48,12.74)	2.91 (0.99,8.61)	0.82(−0.46,2.10)
**Intravaginal cleansing during bathing**	p = 0.408	p = 0.057	p = 0.557	p = 0.178	p = 0.099
***No***	Ref	Ref	Ref	Ref	Ref
***Yes***	0.81(0.50,1.32	1.58 (0.99,2.53)	0.88(0.58,1.34)	0.75(0.50,1.14)	0.44 (−0.08,0.96)
**Contraception use**	p = 0.224	p = 0.445	p = 0.293	p = 0.373	p = 0.125
***None/Non-Hormonal***	Ref	Ref	Ref	Ref	Ref
***Hormonal***	0.70(0.41,1.22)	0.78(0.47,1.31)	0.83(0.52,1.30)	0.83(0.52,1.32)	−0.53(−1.09,0.04)
**Recent antibiotic use**	p = 0.110	p = 0.606	p = **0.054**	p = **0.052**	p = 0.832
***No***	Ref	Ref	Ref	Ref	Ref
***Yes***	0.63(0.33,1.21)	0.65(0.38,1.13)	1.76 (0.98,3.15)	1.74 (1.00,3.03)	−0.64(−1.26,-0.01)

Results that remained significant (using a p = 0.05 cut-off) in the model are depicted in bold. *: non- logistic regression model. qPCR: quantitative polymerase chain reaction. ^1^Data collected at the screening visit up to two months before the enrolment visit. Nr: number. The numbers in each category for the variables are listed in the Additional file 2.

## Discussion

This study characterised key microbiota in the female genital tract, and compared their presence and concentrations among healthy women at average risk to those at high risk of HIV infection in three sites in sub-Saharan Africa. The presence of species and *Lactobacillus* genus across Kenyan, South African and Rwandan women was remarkably similar and few differences were seen between the two large groups of reference women in Kenya and South Africa. However, the sex workers and HIV-positive women from Rwanda had the highest *G. vaginalis* presence, and pregnant women had a higher *Lactobacillus* concentration. Additionally, this study found that recent sexual exposure negatively affected the presence of *L. crispatus*, *L. vaginalis*, and *Lactobacillus* genus, and that having more than one sexual partner in the last three months was strongly associated with an increased presence of *G. vaginalis* and *L. iners*.

This is the first study showing that, in addition to *L. crispatus, L. vaginalis* may play an important role in the health of the vaginal microbiota in African women. *L. vaginalis* together with *L. crispatus* showed the strongest association (p < 0.001) with a healthy Nugent score in the reference group (i.e. adult, non-pregnant, non-sex worker women) and in all women combined. *L. vaginalis* was present in concentrations above 10^6^ geq/ml for 36% of the 28% of women with detectable *L. vaginalis*. We previously demonstrated a *L. vaginalis* presence of 73% in healthy and of 8% in women with BV in a study in Belgium [13]. A recent study that characterized the microbiota among seven women in the US by 16S rRNA sequencing identified *L. vaginalis*, in 3 out of 7 healthy women; however, *L. vaginalis* contributed to less than 0.05% of the communities [28]. In a study from Burkina Faso, *L. vaginalis* represented only 0.5% of sequences in a cluster of 30 participants with dominant genus *Lactobacillus,* compared to 77% for *L. iners* and 11% for *L. crispatus* [4]. In a study that compared vaginal microbiota between unspecified adult populations in Uganda and Korea, *L. vaginalis*, detected by 16S rRNA after isolation on *Lactobacillus* Rogosa SL agar, was common in Uganda and absent in Korea, whereas *L. crispatus* was common in both populations [29]. There is also mention of the presence of *L. vaginalis*, detected by Randomly Amplified Polymorphic DNA and 16S rRNA after isolation on Rogosa agar, in a small Swedish study of 20 healthy women in the vagina as well as the rectum [30]. More research is needed to understand the role and function of *L. vaginalis*, in addition to other *Lactobacillus* species prevalent in the vaginal niche. Mendez-Soares and colleagues performed functional genomics of 25 species of vaginal, gastrointestinal and food product lactobacilli [31]. They described differences in genes encoding for proteins that interact with the host, as well as other bacteria, between *L. crispatus, L. iners, L. jensenii, L. gasseri*. This suggests that these species have different mechanisms for interacting with their environment. Also, it has been shown that different growth limiting factors exist between species. Boskey and colleagues showed *in vitro* that the growth limiting factor for *L. vaginalis* was a depletion of a metabolite or the buildup of an unspecified toxic waste product; this is in contrast to *L. crispatus* and *L. gasseri* in which the growth limiting factor is the lowest point of acidity reached due to species lactic acid production [32].

While several published papers confirm that lactobacilli are the dominant species in a healthy vaginal environment and are replaced by other commensal anaerobes (but remain present in very low numbers) in women with BV, there is a paucity of qPCR data on *Lactobacillus* genus and species in African populations. Individual species have been characterised in several settings in the US, Europe, Australia and Asia, yet we could identify only three papers for sub-Saharan Africa presenting quantitative data: two from East and one from West Africa. In Niger, 241 asymptomatic women attending a health care clinic were included [33]. *Lactobacillus* genus was absent in 34 women with a diagnosis of BV by Nugent score (14.2% of total women). HIV-positive women in Kenya had a *L. crispatus* mean log concentration just below 6 (copies/swab) which is higher than the 4.7 log (geq/ml) that we observed in the HIV-positive women in Rwanda [34]*.* Benning and colleagues retrospectively sequenced the 16S rRNA gene in 40 cervicovaginal lavage samples from a Rwandan cohort that included HIV-positive women [35]. Compared to our data in the HIV-positive women, they detected lower presence of *Lactobacillus* genus (67% vs 80%), *L. crispatus* (11% vs 17%), *L. iners* (50% vs 63%), and *L. vaginalis* (11% vs 30%). These variations in results may be explained by the small sample size and by the difference in methods used. Pregnancy has been shown to be associated with low bacterial diversity and high levels of lactobacilli, particularly *L. crispatus* [36,37]. The higher *Lactobacillus* concentrations that we observed in the pregnant women agrees with a longitudinal study in 22 pregnant, mostly African American women, showing a higher abundance in 16S rRNA V1-V3 of *Lactobacillus vaginalis, L. crispatus, L. gasseri and L. jensenii* as compared to 20 non-pregnant women, of whom 10 were African American [38]. Similarly, a US longitudinal study of 12 Caucasian healthy women showed a stable *Lactobacillus* dominant vaginal microbiome (16S rRNA V3-V5) throughout pregnancy [39]. In future, larger and longitudinal studies are needed to adequately characterise the vaginal microbiota among women in sub-Saharan Africa.

*L. iners* was present in 82% of BV-negative, in 62% of intermediate, and in 67% of BV-positive samples by Nugent score. It is the only *Lactobacillus* species that was present in high concentrations in samples that are also characterised by high concentrations of *A. vaginae* and *G. vaginalis*. In contrast with the other lactobacilli, *L. iners* did not show an association with vaginal pH (p = 0.562) and therefore seems to be resilient to a less acidic BV environment. Further, recent exploratory *in vitro* data showed that *G. vaginalis* displaced *L. crispatus* but not *L. iners* [40]. *L. iners* has been detected as the dominant species in some studies (e.g. in healthy White and Black American women [41]); in combination with *L. crispatus* in Japanese women [42], Chinese women [43], Estonian women [44], and Caucasian Belgian women [13]; or together with multiple *Lactobacillus* species in non-Black American women [41]. *L. iners* has also been shown to be present, though to a lesser extent, in BV-positive samples in other populations (e.g. 6.6% of sequences in Swedish women) [45].

Sexual behaviours were shown to be associated with concentration of vaginal microbiota. PSA, a validated marker of recent unprotected sexual intercourse in the past 72 hours, was strongly associated with a reduced prevalence of *L. crispatus* and *L. vaginalis* and lower concentrations of *Lactobacillus* genus. These data are congruent with a contraceptive study which reported that condom use was associated with higher *L. crispatus* concentrations (+2 log geq/ml, p < 0.001) compared to intra uterine device users or women using the rhythm method [46]. This result supports the hypothesis that the exposure to the alkaline semen alters the vaginal microbiota. We reported an increased presence of *G. vaginalis* and *L. iners* with more than ‘one sexual partner in the last three months’. BV has been shown to be associated with exposure to a new partner in other reports [47,48]. We could not explore this association due to a low number of women reporting a new sex partner. Recently, it has been postulated that certain subgroups of *G. vaginalis* may cause a different clinical outcome [49,50]. Therefore, a new partner could possibly introduce a new *G. vaginalis* strain and lead to microbial instability. In conclusion, we theorise that the combination of an immediate decrease in *Lactobacillus* species initiated by alkaline semen, and the acquisition of a new strain of BV-associated bacteria may lead to incident BV episodes.

We detected a higher presence of *Lactobacillus* species, with the exception of *L. iners*, for women with a low pH. The association of pH with individual vaginal species has not been described previously but a higher abundance of lactobacilli has been associated with a lower vaginal pH among 100 cycling Chinese women [43], 494 asymptomatic Estonian women [44], and 396 asymptomatic North American women [20]. The median pH observed in the reference groups was well above 4.2, the cut-off for which values below are reported as normal [51]. The pH, measured in clinical studies as one of the Amsel criteria, is not often described separately in the literature and data is lacking for women in Africa and in general. A median pH of 3.6 was described in a Belgian healthy population of 141 women [52]. Moreover, the vaginal pH in different ethnic groups in North America was as follows: Hispanic 5.0; black 4.7; Asian 4.4 and white 4.2 [20] and for Estonian women the mean value was 4.7 [44].

To our knowledge, the association of parity with a decrease in *L. vaginalis* and *L. iners* presence has not been described previously. This suggests that pregnancy, a period of high oestrogen status with high lactobacilli presence, is followed by a reduction of certain strains of the lactobacilli species. It is possible that the delivery period could disrupt the stable vaginal lactobacillus population attained during pregnancy. This hypothesis needs further study. *C. albicans* was positively associated with parity in our study. It is known to be associated with pregnancy which may indirectly explain the association with parity. However, a study in 500 Australian pregnant women showed no difference for vaginal candida colonization and parity history [53]. This may indicate that candida colonization does not normalize after delivery.

Though research has shown that intravaginal cleansing is a risk factor for BV [54,55], we did not observe a negative effect of the use of products to externally wash, internally cleanse, or use of products for drying or tightening on the presence or concentrations of species or on BV status by Nugent scoring. On the contrary, in the reference group, a negative association with *A. vaginae* and a positive association with *Lactobacillus* genus were present among those who reported cleansing during bathing. A systematic review of longitudinal studies concluded that intravaginal cleansing with soap was associated with the development of intermediate vaginal flora and bacterial vaginosis in women with normal vaginal flora at baseline (pooled adjusted odds ratio 1.24, 95% CI 1.04–1.47) [55]. However, there is also evidence that vaginal practices are highly heterogeneous and, therefore different study populations use different practices and products [56]. Indeed, women in this study mostly performed washing with water during bathing as opposed to using soap or detergents, which may explain our findings.

Although our data showed a link between the presence of *E. coli* and intermediate BV (p = 0.011) there is yet no evidence that *E. coli* is a regular member of the dysbiotic bacterial community. In a recent review by van de Wijgert and colleagues, only three molecular studies reported vaginal microbiome clusters dominated by streptococci, staphylococci, and/or *E. coli* [43,57,58]. It is possible that the association between *E. coli* and BV is confounded by unprotected sex. A study among 44 East African sex workers showed four types of microbiota of which one was dominated by *E. coli*, mostly present in HIV-positive women, and was distinct from BV [59]. Further, early culture-based studies documented that intercourse led to an increase in vaginal *E. coli* [60]. This theory could explain the high presence of *E. coli* (70%) in the sex worker group in our study. Additionally, the higher presence of *E coli* in the sex workers in our study may indicate sexual practices such as anal sex in addition to perianal contamination during vaginal intercourse [61].

This study has several strengths; sampling from women in three different African countries; sampling populations of women at different risks for STI and HIV; and a quantitative estimation of abundance for well-known dominant organisms of the vaginal microbiota which allowed for extensive profiling of a relatively large number of samples. This study also has several limitations: the sample size of the subgroups was small compared to the large reference groups; the analysis was cross-sectional; multiple testing was performed in defining associations between the variables and species; co-linearity was present e.g. age and parity were correlated (r = 0.65), complicating the interpretation of independent effects. Consequently, causation and effects of single associations should be interpreted cautiously. Furthermore, we excluded correlated variables and we did not correct for multiple testing as this was a hypothesis generating analysis. Importantly, the study did not aim to investigate the function of species or sub-species.

## Conclusion

In conclusion, our study in sub-Saharan women provides important baseline data for several important species related to a healthy and dysbiotic vaginal microbiota. Our q PCR results highlight the similarity of *Lactobacillus* genus and species concentration across two East Africa and one South African site, and between different sub-groups of women. There were also some important differences by pregnancy status and sexual behaviour. Unsurprisingly, we found high concentrations of *L. iners*; but we also found a high concentration of *L. vaginalis* which has not been well described in other reports. We detected an overall lower presence of *Lactobacillus* species compared to Asian, US and European populations. These data may explain some of the increased vulnerability of these populations to STI including HIV. Longitudinal studies are needed to study health outcomes, including acquisition of STI and HIV. Additionally, research is needed to understand the role and function of different microbial species and sub-species (e.g. *G. vaginalis*) in relation to the protection and susceptibility to infection. Ultimately, these data stress the need to invest more research in order to develop novel methods to improve reproductive health and the prevention of infection.

## Additional files

Additional file 1:
**Overview of the quantitative PCR assays.**
Additional file 2:
**Sociodemographic, behavioural, and clinical characteristics by group.**
Additional file 3:
**Vaginal microbiota species qPCR presence and pH by group.**
Additional file 4
**Distribution plots of quantitative PCR data.**

## Abbreviations

AOR: Adjusted odds ratio
BV: Bacterial vaginosis
CI: confidence interval
CT: Chlamydia trachomatis
HSV-2: herpes simplex virus type 2
KE: Kenya
NG: Neisseria gonorrhoea
PBS: Phosphate buffered saline
PCR: Polymerase chain reaction
PSA: Prostate-specific antigen
qPCR: quantitative polymerase chain reaction
RTI: Reproductive tract infections
RW: Rwanda
SA: South Africa
STI: Sexually transmitted infections
SD: Standard deviation
TV: Trichomonas vaginalis
