# Supplementary material for: The significance of Lactobacillus crispatus and L. vaginalis for vaginal health and the negative effect of recent sex: a cross-sectional descriptive study across groups of African women
Source: BMC Infect Dis. 2015 Mar 4;15:115. doi: 10.1186/s12879-015-0825-z (PMC4351943; doi:10.1186/s12879-015-0825-z)
Supplement: Additional file 1: — Overview of the quantitative PCR assays. [file 12879_2015_825_MOESM1_ESM.docx]

**Additional file 1 Overview of the quantitative PCR assays**

| **qPCR assay** | **Reference** | **Primers sequence 5’ – 3’** | **Target gene** | **Cycling conditions** | **Primer**  **concentration** |
| --- | --- | --- | --- | --- | --- |
| *Atopobium vaginae* | [1] | AV-F: CCCTATCCGCTCCTGATACC  AV-R: CCAAATATCTGCGCATTTCA | 16S rRNA | 10 min 95 °C, 40 (15s 95 °C, 20s 64 °C, 25s 72 °C) | 700 nM  700 nM |
| *Gardnerella vaginalis* | [2] | F-GV1: TTACTGGTGTATCACTGTAAGG  R-GV3: CCGTCACAGGCTGAACAGT | 16S rRNA | 10 min 95 °C, 40 (45s 95 °C, 45s 55 °C, 45s 72 °C) | 1250 nM  625 nM |
| *Candida albicans* | [3] | CA_rRNA_f: TTTGCTTGAAAGACGGTA^*^  CA_rRNA_r: TTGAAGATATACGTGGTGG^*^ | ITS-1 | 10 min 95 °C, 45 (15s 95 °C, 60s 60 °C) | 250 nM  250 nM |
| *Prevotella bivia* | [4] | PBsulF: ACGTTTGGGCAAAGCTCCTTGTCT  PBsulR: GCGTGTACGCCAGTTGCAAGA | mucin-desulfating sulfatase | 1 min 95 °C, 40 (15s 94 °C, 40s 58 °C, 30s 72 °C) | 200 nM  200 nM |
| *Escherichia coli* | [5] | EcoliFW: CAACGAACTGAACTGGCAGA  EcoliRV: CATTACGCTGCGATGGAT | *uidA* | 2 min 50 °C, 10 min 95 °C, 40 (15s 95 °C, 60s 60 °C) | 300 nM  300 nM |
| *Lactobacillus* genus | [2] | LBF:ATGGAAGAACACCAGTGGCG  LBR: CAGCACTGAGAGGCGGAAAC | 16S rRNA | 15 min 95 °C, 37 (15s 95 °C, 45s 50 °C, 45s 72 °C) | 150 nM  150 nM |
| *Lactobacillus crispatus* | [6] | LcrisF: AGCGAGCGGAACTAACAGATTTAC LcrisR : AGCTGATCATGCGATCTGCTT | 16S rRNA | 15 min, 95 °C, 40 (15s 95 °C, 60s 60 °C, 20s 72 °C) | 100 nM  100 nM |
| *Lactobacillus gasseri* | [7] | LgassF: AGCGAGCTTGCCTAGATGAATTTG LgassR:TCTTTTAAACTCTAGACATGCGTC | 16S rRNA | 15 min 95 °C, 40 (15s 95 °C, 60s 57 °C, 60s 65 °C) | 200 nM  200 nM |
| *Lactobacillus iners* | [8] | InersFw: GTCTGCCTTGAAGATCGG  InersRev: ACAGTTGATAGGCATCATC | 16S rRNA | 15 min 95 °C, 35 (15s 95 °C, 55s 60 °C, 60s 65 °C) | 200 nM  200 nM |
| *Lactobacillus jensenii* | [7] | LjensF:AAGTCGAGCGAGCTTGCCTATAGA  LjensR: CTTCTTTCATGCGAAAGTAGC | 16S rRNA | 15 min 95 °C, 40 (15s 95 °C, 55s 60 °C, 60s 72 °C) | 300 nM  300 nM |
| *Lactobacillus vaginalis* | [9] | LV16s_23s_F:GCCTAACCATTTGGAGGG  LV16s_23s_R3: CGATGTGTAGGTTTCCG | 16S-23S rRNA | 15 min 95 °C, 37 (15s 95 °C, 30s 56 °C, 30s 72 °C) | 200 nM  200 nM |

*: adapted from reference 3 by Guiver et al.

Reference List

1. Menard JP, Fenollar F, Raoult D, Boubli L, Bretelle F. Self-collected vaginal swabs for the quantitative real-time polymerase chain reaction assay of Atopobium vaginae and Gardnerella vaginalis and the diagnosis of bacterial vaginosis. *Eur J Clin Microbiol Infect Dis* 2011.

2. Zariffard MR, Saifuddin M, Sha BE, Spear GT. Detection of bacterial vaginosis-related organisms by real-time PCR for Lactobacilli, Gardnerella vaginalis and Mycoplasma hominis. *FEMS Immunol Med Microbiol* 2002; **34(4)**:277-281.

3. Guiver M, Levi K, Oppenheim BA. Rapid identification of candida species by TaqMan PCR. *J Clin Pathol* 2001; **54(5)**:362-366.

4. Lopes dos Santos Santiago G, Tency I, Verstraelen H, Verhelst R, Trog M, Temmerman M*, et al.* Longitudinal qPCR study of the dynamics of L. crispatus, L. iners, A. vaginae, (sialidase positive) G. vaginalis, and P. bivia in the vagina. *PLoS One* 2012; **7(9)**:e45281.

5. Chern EC, Siefring S, Paar J, Doolittle M, Haugland RA. Comparison of quantitative PCR assays for Escherichia coli targeting ribosomal RNA and single copy genes. *Lett Appl Microbiol* 2011; **52(3)**:298-306.

6. Byun R, Nadkarni MA, Chhour KL, Martin FE, Jacques NA, Hunter N. Quantitative analysis of diverse Lactobacillus species present in advanced dental caries. *J Clin Microbiol* 2004; **42(7)**:3128-3136.

7. Tamrakar R, Yamada T, Furuta I, Cho K, Morikawa M, Yamada H*, et al.* Association between Lactobacillus species and bacterial vaginosis-related bacteria, and bacterial vaginosis scores in pregnant Japanese women. *BMC Infect Dis* 2007; **7**:128.

8. De Backer E, Verhelst R, Verstraelen H, Alqumber MA, Burton JP, Tagg JR*, et al.* Quantitative determination by real-time PCR of four vaginal Lactobacillus species, Gardnerella vaginalis and Atopobium vaginae indicates an inverse relationship between L. gasseri and L. iners. *BMC Microbiol* 2007; **7(1)**:115.

9. Jespers V, Menten J, Smet H, Poradosu S, Abdellati S, Verhelst R*, et al.* Quantification of bacterial species of the vaginal microbiome in different groups of women, using nucleic acid amplification tests. *BMC Microbiol* 2012; **12**:83.
