# Supplementary material for: The significance of Lactobacillus crispatus and L. vaginalis for vaginal health and the negative effect of recent sex: a cross-sectional descriptive study across groups of African women
Source: BMC Infect Dis. 2015 Mar 4;15:115. doi: 10.1186/s12879-015-0825-z (PMC4351943; doi:10.1186/s12879-015-0825-z)
Supplement: Additional file 2: — Sociodemographic, behavioural, and clinical characteristics by group. [file 12879_2015_825_MOESM2_ESM.docx]

**Additional file 2 Vaginal microbiota species qPCR presence and pH by group**

|  | **Reference group** | | **Pregnant women** | | **Adolescents** | | **Intra-vaginal practices** | **Sex workers** | **HIV-positive** |
| --- | --- | --- | --- | --- | --- | --- | --- | --- | --- |
|  | Kenya  N=109 | South Africa  N=108 | Kenya  N=30 | South Africa  N=30 | Kenya  N=29 | South Africa  N=30 | South Africa  N=30 | Rwanda  N=30 | Rwanda  N=30 |
| ***Lactobacillus* genus** | N (%) | N (%) | N (%) | N (%) | N (%) | N (%) | N (%) | N (%) | N (%) |
| Absent | 8(7) | 10(9) | 0 | 0 | 2(7) | 6(20) | 2(7) | 2(7) | 6(20) |
| Present, not quantifiable | 10(9) | 5(5) | 0 | 3(10) | 0 | 5(17) | 3(10) | 1(3) | 3(10) |
| < 1.000.000 geq/ml | 18(17) | 14(13) | 2(7) | 8(27) | 3(10) | 7(23) | 4(13) | 3(10) | 5(17) |
| >= 1.000.000 geq/ml | 73(67) | 79(73) | 28(93) | 19(63) | 24(83) | 12(40) | 21(70) | 24(80) | 16(53) |
| ***L. crispatus*** |  |  |  |  |  |  |  |  |  |
| Absent | 80(73) | 82(76) | 24(80) | 23(77) | 18(62) | 24(80) | 25(83) | 25(83) | 25(83) |
| Present, not quantifiable | 3(3) | 1(1) | 0 | 0 | 1(3) | 0 | 0 | 0 | 2(7) |
| < 1.000.000 geq/ml | 1(1) | 0 | 0 | 1(3) | 0 | 2(7) | 1(3) | 1(3) | 1(3) |
| >= 1.000.000 geq/ml | 25(23) | 25(23) | 6(20) | 6(20) | 10(34) | 4(13) | 4(13) | 4(13) | 2(7) |
| ***L. iners*** |  |  |  |  |  |  |  |  |  |
| Absent | 34(31) | 26(24) | 6(20) | 7(23) | 6(21) | 10(33) | 4(13) | 12(40) | 11(37) |
| Present, not quantifiable | 8(7) | 6(6) | 0 | 1(3) | 1(3) | 7(23) | 3(10) | 0 | 2(7) |
| < 1.000.000 geq/ml | 12(11) | 8(7) | 2(7) | 3(10) | 2(7) | 1(3) | 4(13) | 0 | 1(3) |
| >= 1.000.000 geq/ml | 55(50) | 68(63) | 22(73) | 19(63) | 20(69) | 12(40) | 19(63) | 18(60) | 16(53) |
| ***L. jensenii*** |  |  |  |  |  |  |  |  |  |
| Absent | 90(83) | 83(77) | 24(80) | 20(67) | 22(76) | 28(93) | 24(80) | 29(97) | 27(90) |
| Present, not quantifiable | 4(4) | 2(2) | 0 | 0 | 2(7) | 1(3.5) | 2(7) | 0 | 0 |
| < 1.000.000 geq/ml | 2(2) | 6(6) | 1(3) | 1(3) | 0 | 0 | 1(3) | 0 | 1(3) |
| >= 1.000.000 geq/ml | 13(12) | 17(16) | 5(17) | 9(30) | 5(17) | 1(3.5) | 3(10) | 1(3) | 2(7) |
| ***L. gasseri*** |  |  |  |  |  |  |  |  |  |
| Absent | 102(94) | 100(93) | 27(90) | 27(90) | 25(86) | 28(93) | 28(93) | 26(87) | 27(90) |
| Present, not quantifiable | 4(4) | 1(1) | 0 | 1(3) | 1(3) | 0 | 1(3) | 2(7) | 3(10) |
| < 1.000.000 geq/ml | 1(1) | 4(4) | 2(7) | 1(3) | 2(7) | 0 | 0 | 0 | 0 |
| >= 1.000.000 geq/ml | 2(2) | 3(3) | 1(3) | 1(3) | 1(3) | 2(7) | 1(3) | 2(7) | 0 |
| ***L. vaginalis*** |  |  |  |  |  |  |  |  |  |
| Absent | 76(70) | 80(74) | 21(70) | 23(77) | 15(52) | 23(77) | 25(83) | 22(73) | 21(70) |
| Present, not quantifiable | 6(6) | 3(3) | 1(3) | 3(10) | 3(10) | 2(7) | 2(7) | 0 | 2(7) |
| < 1.000.000 geq/ml | 18(17) | 15(14) | 6(20) | 1(3) | 6(21) | 4(13) | 2(7) | 0 | 2(7) |
| >= 1.000.000 geq/ml | 9(8) | 10(9) | 2(7) | 3(10) | 5(17) | 1(3) | 1(3) | 8(27) | 5(17) |
|  | **Reference group** | | **Pregnant women** | | **Adolescents** | | **Intra-vaginal practices** | **Sex workers** | **HIV-positive** |
|  | Kenya  N=109 | South Africa  N=108 | Kenya  N=30 | South Africa  N=30 | Kenya  N=29 | South Africa  N=30 | South Africa  N=30 | Rwanda  N=30 | Rwanda  N=30 |
| ***G. vaginalis*** |  |  |  |  |  |  |  |  |  |
| Absent | 50(46) | 59(55) | 16(53) | 15(50) | 12(41) | 11(37) | 14(47) | 7(23) | 9(30) |
| Present, not quantifiable | 0 | 0 | 0 | 0 | 0 | 1(3) | 1(3) | 0 | 0 |
| < 1.000.000 geq/ml | 40(37) | 37(34) | 6(20) | 12(40) | 12(41) | 17(57) | 12(40) | 8(27) | 11(37) |
| >= 1.000.000 geq/ml | 19(17) | 12(11) | 8(27) | 3(10) | 5(17) | 1(3) | 3(10) | 15(50) | 10(33) |
| ***A. vaginae*** |  |  |  |  |  |  |  |  |  |
| Absent | 63(58) | 71(66) | 21(70) | 19(63) | 19(66) | 11(37) | 19(63) | 13(43) | 16(53) |
| Present, not quantifiable | 3(3) | 0 | 0 | 0 | 2(7) | 0 | 0 | 1(3) | 1(3) |
| < 1.000.000 geq/ml | 21(19) | 26(24) | 1(3) | 9(30) | 1(3) | 14(47) | 7(23) | 4(13) | 8(27) |
| >= 1.000.000 geq/ml | 22(20) | 11(10) | 8(27) | 2(7) | 7(24) | 5(17) | 4(14) | 12(40) | 5(17) |
| ***P. bivia*** |  |  |  |  |  |  |  |  |  |
| Absent | 12(11) | 25(23) | 1(3) | 9(30) | 5(17) | 18(60) | 8(27) | 0 | 9(30) |
| Present, not quantifiable | 12(11) | 0 | 3(10) | 0 | 2(7) | 0 | 0 | 1(3) | 0 |
| < 1.000 geq/ml | 31(28) | 42(39) | 18(60) | 9(30) | 9(31) | 6(20) | 9(30) | 7(23) | 10(33) |
| >= 1.000 geq/ml | 54(50) | 41(38) | 8(27) | 12(40) | 13(45) | 6(20) | 13(43) | 22(73) | 11(37) |
| ***E. coli*** |  |  |  |  |  |  |  |  |  |
| Absent | 81(74) | 79(73) | 24(80) | 20(67) | 21(72) | 26(87) | 21(70) | 9(30) | 24(80) |
| < 1.000.000 geq/ml | 27(25) | 27(25) | 6(20) | 9(30) | 8(28) | 4(13) | 9(30) | 19(63) | 5(17) |
| >= 1.000.000 geq/ml | 1(1) | 2(2) | 0 | 1(3) | 0 | 0 | 0 | 2(7) | 1(3) |
| ***C. albicans*** |  |  |  |  |  |  |  |  |  |
| Absent | 94(86) | 96(89) | 26(87) | 29(97) | 26(90) | 30(100) | 25(83) | 26(87) | 27(90) |
| Present, not quantifiable | 1(1) | 0 | 0 | 0 | 0 | 0 | 0 | 0 | 0 |
| < 1.000.000 geq/ml | 12(11) | 10(9) | 3(10) | 1(3) | 2(7) | 0 | 4(13) | 3(10) | 3(10) |
| >= 1.000.000 geq/ml | 2(2) | 2(2) | 1(3) | 0 | 1(3) | 0 | 1(3) | 0 | 0 |
| ***Vaginal pH-category*** | N (%) | N (%) | N (%) | N (%) | N (%) | N (%) | N (%) | N (%) | N (%) |
| <4 | 4(4) | 18(17) | 3(10) | 5(17) | 4(14) | 4(14) | 3(10) | 0 | 2(7) |
| 4-4.5 | 25(23) | 41(38) | 10(33) | 19(63) | 8(28) | 14(48) | 10(33) | 10(33) | 9(30) |
| 4.6-5 | 44(40) | 39(36) | 7(23) | 5(17) | 13(45) | 8(28) | 12(40) | 8(27) | 10(33) |
| 5.1-5.5 | 9(8) | 7(6) | 4(13) | 1(3) | 0 | 2(7) | 1(3) | 8(27) | 7(23) |
| >5.5 | 27(25) | 3(3) | 6(20) | 0 | 4(14) | 1(3) | 4(13) | 4(13) | 2(7) |

Quantitative PCR data was available for 426of the 430 women. qPCR: quantitative polymerase chain reaction.
