# Supplementary material for: The significance of Lactobacillus crispatus and L. vaginalis for vaginal health and the negative effect of recent sex: a cross-sectional descriptive study across groups of African women
Source: BMC Infect Dis. 2015 Mar 4;15:115. doi: 10.1186/s12879-015-0825-z (PMC4351943; doi:10.1186/s12879-015-0825-z)
Supplement: Additional file 3: — Vaginal microbiota species qPCR presence and pH by group. [file 12879_2015_825_MOESM3_ESM.docx]

**Additional file 3 Sociodemographic, behavioural, and clinical characteristics by group**

|  | **Reference group** | | **Pregnant women^1^** | | **Adolescents** | | **Intra-vaginal practices^2^** | **Sex workers** | **HIV-positive^3^** |
| --- | --- | --- | --- | --- | --- | --- | --- | --- | --- |
|  | **Kenya**  **N=110** | **South Africa**  **N=109** | **Kenya**  **N=30** | **South Africa**  **N=30** | **Kenya**  **N=30** | **South Africa**  **N=30** | **South Africa**  **N=31** | **Rwanda**  **N=30** | **Rwanda**  **N=30** |
|  | N (%)^4^ | N (%)^4^ | N (%)^4^ | N (%)^4^ | N (%)^4^ | N (%)^4^ | N (%)^4^ | N (%)^4^ | N (%)^4^ |
| ***Age in years*** |  |  |  |  |  |  |  |  |  |
| **Mean (range)** | 25(18-35) | 25(18-34) | 26(19-34) | 25(18-40) | 16(16-17) | 16(16-17) | 24(19-33) | 27(22-33) | 31(22-35) |
| ***Parity*** |  |  |  |  |  |  |  |  |  |
| **0** | 24(22) | 34(31) | 11(37) | 20(67) | 24(80) | 27(90) | 5(16) | 1(3) | 3(10) |
| **1-2** | 62(56) | 60(55) | 15(50) | 9(30) | 6(20) | 3(10) | 24(78) | 21(70) | 17(57) |
| **>2** | 24(22) | 15(14) | 4(13) | 1(3) | 0 | 0 | 2(6) | 8(27) | 10(33) |
| ***Lifetime number of sex partners*** |  |  |  |  |  |  |  |  |  |
| **1** | 32(29) | 18(17) | 12(40) | 6(20) | 19(63) | 18(60) | 5(16) | 0 | 2(7) |
| **2-3** | 63(57) | 50(46) | 15(50) | 17(57) | 6(20) | 11(37) | 13(42) | 0 | 16(53) |
| **>3** | 15(14) | 41(38) | 3(10) | 7(23) | 5(17) | 1(3) | 13(42) | 30(100) | 12(40) |
| ***Number of sex partners last 3 months ^4^*** |  |  |  |  |  |  |  |  |  |
| **0** | 9(8) | 7(6) | 0 | 1(3) | 4(13) | 2(7) | 1(3) | 0 | 2(7) |
| **1** | 99(90) | 99(91) | 30(100) | 28(93) | 26(87) | 25(83) | 27(87) | 1(3) | 24(80) |
| **>1** | 2(2) | 3(3) | 0 | 1(3) | 0 | 3(10) | 3(10) | 29(97) | 4(13) |
| ***Reported recent vaginal sex at enrolment ^5^*** | 19(17) | 17(16) | 10(33) | 3(10) | 2(7) | 0 | 4(13) | 16(53) | 11(37) |
| ***Seminal factor PSA present at enrolment ^6^*** | 25(23) | 58(55) | 16(53) | 16(59) | 5(17) | 16(59) | 14(45) | 17(57) | 16(53) |
| ***Colposcopic findings*** |  |  |  |  |  |  |  |  |  |
| **Yes** | 4(4) | 19(18) | 1(3) | 1(3) | 1(3) | 3(10) | 3(10) | 7(23) | 4(13) |
| **Abrasion** | 0 | 1(1) | 0 | 1(3) | 0 | 0 | 2(6) | 1(3) | 0 |
| **Edema** | 0 | 0 | 0 | 0 | 0 | 0 | 0 | 0 | 1(3) |
| **Ecchymosis** | 0 | 4(4) | 0 | 1(3) | 0 | 0 | 0 | 0 | 0 |
| **Erythema** | 3(3) | 0 | 1(3) | 0 | 0 | 1(3) | 1(3) | 3(10) | 1(3) |
| **Laceration** | 0 | 2(2) | 1(3) | 0 | 0 | 1(3) | 0 | 0 | 0 |
| **Peeling** | 0 | 0 | 0 | 0 | 0 | 0 | 0 | 0 | 0 |
| **Petechiae** | 0 | 15(14) | 0 | 0 | 0 | 1(3) | 0 | 1(3) | 3(10) |
| **Ulcer** | 1(1) | 3(3) | 0 | 0 | 1(3) | 0 | 0 | 1(3) | 0 |
|  | **Reference group** | | **Pregnant women^1^** | | **Adolescents** | | **Intra-vaginal practices^2^** | **Sex workers** | **HIV-positive^3^** |
|  | **Kenya**  **N=110** | **South Africa**  **N=109** | **Kenya**  **N=30** | **South Africa**  **N=30** | **Kenya**  **N=30** | **South Africa**  **N=30** | **South Africa**  **N=31** | **Rwanda**  **N=30** | **Rwanda**  **N=30** |
| ***Degree of cervical ectopy*** |  |  |  |  |  |  |  |  |  |
| **Absent** | 75(68) | 46(43) | 24(80) | 19(63) | 23(77) | 18(60) | 13(42) | 6(20) | 5(17) |
| **Small** | 10(9) | 15(14) | 2(7) | 1(3) | 0 | 1(3) | 5(16) | 10(33) | 10(33) |
| **Moderate** | 22(20) | 45(42) | 4(13) | 10(33) | 7(23) | 11(37) | 13(42) | 14(47) | 15(50) |
| **Large** | 3(3) | 2(2) | 0 | 0 | 0 | 0 | 0 | 0 | 0 |
| ***Reproductive tract infections at screening ^7^*** |  |  |  |  |  |  |  |  |  |
| **Herpes simplex virus 2** | 31(28) | 44(40) | 5(17) | 11(37) | 3(10) | 1(3) | 14(45) | 14(47) | 24(83) |
| **Syphilis** | 0 | 0 | 0 | 1(3) | 0 | 0 | 0 | 2(7) | 6(20) |
| **Neisseria gonorrhoea** | 1(1) | 1(1) | 0 | 0 | 0 | 0 | 1(3) | 2(7) | 4(13) |
| **Chlamydia trachomatis** | 4(4) | 18(17) | 2(7) | 4(13) | 0 | 4(13) | 8(26) | 3(10) | 0 |
| **Trichomonas vaginalis** | 3(3) | 5(5) | 1(3) | 3(10) | 4(13) | 0 | 4(14) | 3(10) | 3(10) |
| **Candida species** | 13(12) | 29(27) | 7(23) | 17(57) | 1(3) | 11(37) | 10(32) | 3(10) | 4(13) |
| ***Cleansing inside vagina when bathing*** | 66(60) | 37(34) | 11(37) | 6(20) | 13(43) | 9(30) | 28(90) | 18(60) | 13(43) |
| ***Products used in vagina*** |  |  |  |  |  |  |  |  |  |
| **None** | 35(32) | 35(32) | 17(57) | 13(44) | 16(54) | 16(53) | 0 | 10(33) | 12(40) |
| **Water/fingers only** | 26(24) | 51(47) | 3(10) | 13(43) | 4(13) | 8(27) | 0 | 17(57) | 14(47) |
| **Water and soaps** | 31(28) | 23(21) | 7(23) | 2(7) | 7(23) | 4(13) | 1(3) | 3(10) | 1(3) |
| **Cloth** | 16(15) | 0 | 2(7) | 1(3) | 2(7) | 2(7) | 23(74) | 0 | 3(10) |
| **Lemon juice/detergents** | 2(2) | 0 | 1(3) | 1(3) | 1(3) | 0 | 7(23) | 0 | 0 |
| ***Contraceptive use*** |  |  |  | |  |  |  |  |  |
| **None** | 25(23) | 18(17) | Not Applicable | | 14(47) | 7(23) | 4(13) | 2(7) | 5(17) |
| **Condoms only** | 24(22) | 28(26) |  |  | 12(40) | 18(60) | 11(35) | 7(23) | 9(30) |
| **Combined hormones** | 20(18) | 18(17) |  |  | 1(3) | 2(7) | 5(16) | 3(10) | 2(7) |
| **Progestin-only injectable** | 37(34) | 42(39) |  |  | 3(10) | 3(10) | 11(36) | 18(60) | 11(37) |
| **Intrauterine device** | 4(4) | 2(2) |  |  | 0 | 0 | 0 | 0 | 3(10) |
| **Sterilisation** | 0 | 1(1) |  |  | 0 | 0 | 0 | 0 | 0 |
| ***New systemic antibiotic use 14 days before enrolment*** | 11(10) | 19(17) | 5(17) | 7(23) | 4(13) | 4(13) | 4(13) | 4(13) | 4(13) |
|  | **Reference group** | | **Pregnant women^1^** | | **Adolescents** | | **Intra-vaginal practices^2^** | **Sex workers** | **HIV-positive^3^** |
|  | **Kenya**  **N=110** | **South Africa**  **N=109** | **Kenya**  **N=30** | **South Africa**  **N=30** | **Kenya**  **N=30** | **South Africa**  **N=30** | **South Africa**  **N=31** | **Rwanda**  **N=30** | **Rwanda**  **N=30** |
| ***pH*** |  |  |  |  |  |  |  |  |  |
| **<4** | 4(4) | 18(17) | 3(10) | 5(17) | 4(13) | 4(14) | 3(10) | 0 | 2(7) |
| **4-4.5** | 25(23) | 41(38) | 10(33) | 19(63) | 9(30) | 14(48) | 10(32) | 10(33) | 9(30) |
| **4.6-5** | 44(40) | 40(37) | 7(23) | 5(17) | 13(43) | 8(28) | 13(42) | 8(27) | 10(33) |
| **5.1-5.5** | 10(9) | 7(6) | 4(13) | 1(3) | 0 | 2(7) | 1(3) | 8(27) | 7(23) |
| **>5.5** | 27(25) | 3(3) | 6(20) | 0 | 4(13) | 1(3) | 4(13) | 4(13) | 2(7) |

^1^ Pregnant: up to 14 weeks gestational age at enrolment as defined by abdominal ultrasound; ^2^ Intravaginal Practices: using other substances than the use of water, soap and/or fingers alone; ^3^ HIV-positive women: on antiretroviral treatment for at least 6 months, currently asymptomatic, and a CD4 count of more than 350 cells/µl. ^4^  Last 3 months prior to screening visit; ^5^ Recent vaginal sex: vaginal sex this morning and/or yesterday evening before enrolment visit; ^6^ Prostate specific antigen present in vaginal fluid, including weak reaction. ^7^ Data for *Trichomonas vaginalis* testing was unavailable for 8 women, HSV-2 for one.
