# Supplementary material for: The significance of Lactobacillus crispatus and L. vaginalis for vaginal health and the negative effect of recent sex: a cross-sectional descriptive study across groups of African women
Source: BMC Infect Dis. 2015 Mar 4;15:115. doi: 10.1186/s12879-015-0825-z (PMC4351943; doi:10.1186/s12879-015-0825-z)
Supplement: Additional file 4 — Distribution plots of quantitative PCR data. [file 12879_2015_825_MOESM4_ESM.docx]

**Additional file 4 Distribution plots of quantitative PCR data**

‘Total *Lactobacillus*’ measured by *Lactobacillus* genus.
